# Audit of transvaginal sonography of normal postmenopausal ovaries by sonographers from the United Kingdom Collaborative Trial of Ovarian Cancer Screening (UKCTOCS)

**DOI:** 10.12688/f1000research.15663.1

**Published:** 2018-08-10

**Authors:** Will Stott, Aleksandra Gentry-Maharaj, Andy Ryan, Nazar Amso, Mourad Seif, Chris Jones, Ian Jacobs, Max Parmar, Usha Menon, Stuart Campbell, Matthew Burnell

**Affiliations:** 1 Gynaecological Oncology, UCL EGA Institute of Women's Health, London, WC1E 6BT, UK; 2 Department of Obstetrics and Gynaecology, School of Medicine, Cardiff University, Cardiff, CF14 4XN, UK; 3 Academic Unit of Obstetrics and Gynaecology, St. Mary’s Hospital, Manchester, UK; 4 University of New South Wales, Sydney, NSW, 2052, Australia; 5 Medical Research Council Clinical Trials Unit, University College London, London, WC1V 6LJ, UK; 6 Create Fertility, London, EC2V 6ET, UK

**Keywords:** Ovarian Cancer Screening, Transvaginal Sonography Scans (TVS), Ultrasound, Audit, Quality Control (QC), Visualisation Rate (VR)

## Abstract

**Background:** We report on a unique audit of seven sonographers self-reporting high visualization rates of normal postmenopausal ovaries in the United Kingdom Collaborative Trial of Ovarian Cancer Screening (UKCTOCS). ****This audit was ordered by the trial’s Ultrasound Management Subcommittee after an initiative taken in 2008 to improve the quality of scanning and the subsequent increase in the number of sonographers claiming very high ovary visualisation rates.

**Methods:** Seven sonographers reporting high rates (>89%) of visualizing normal postmenopausal ovaries in examinations performed between 1 ^st^ January and 31 ^st^ December 2008 were identified. Eight experts in gynaecological scanning reviewed a random selection of exams performed by these sonographers and assessed whether visualization of both ovaries could be confirmed (cVR-Both) in the examinations. A random effects bivariate probit model was fitted to analyse the results. **
** **

**Results:** The eight experts reviewed images from 357 examinations performed on 349 postmenopausal women (mean age 60.0 years, range 50.2-73.3) by the seven sonographers. The mean cVR-Both obtained from the model for these sonographers was 67.2% with a range of 47.6-86.5% (95%CI 63.9-70.5%). The range of cVR-Both between the experts was 47.3-88.3% and the intra-class correlation coefficient (ICC) for left and right ovary confirmation was 0.39.
*  *

**Conclusions:** The audit suggests that self-reported visualization of postmenopausal ovaries is unreliable, as visualisation of both ovaries could not be confirmed in almost a third of examinations. The agreement for visualization of both ovaries based on review of a static image between experts and sonographers and between expert reviewers alone was only moderate. Further research is needed to develop reliable Quality Control metrics for transvaginal ultrasound.

## Introduction

The normal ovary of a postmenopausal woman is a small structure (mean volume 1.25ml ^1^) usually situated lateral to the uterine fundus and in close relation to the internal iliac vein. In as many as 40% of transvaginal ultrasound (TVS) examinations ^2^ the ovary may not been seen as typically they shrink with age and are sometimes very difficult to locate ^3, 4^. For this reason in the United Kingdom Collaborative Trial of Ovarian Cancer Screening (UKCTOCS) and other screening trials ^2, 5, 6^ a pragmatic approach is taken whereby an annual screening examination may be judged satisfactory even if both ovaries are not seen, given that a good view has been achieved of the Iliac vessels in the pelvic side wall. However, the sonographer should always attempt to visualize both ovaries as this provides the maximum assurance that an early ovarian cancer has been excluded.

A metric commonly used in the quality control (QC) of TVS is self-reported visualisation rate (VR), defined as the number of examinations in which the ovaries were visualized as a proportion of all examinations performed by the sonographer ^7^. In 2008, UKCTOCS implemented an accreditation programme which included the monitoring of individual sonographer VR over a 3 month period ^8^. This revealed that some sonographers were self-reporting higher than expected VR. Therefore in 2009, it was decided to audit the performance of these high scoring sonographers to confirm independently whether it is possible to achieve high rates of ovary visualisation in postmenopausal women. We report on this audit and its outcome.

## Methods

### UKCTOCS trial

The TVS in this study were performed as part of the UKCTOCS, which is a multi-centre randomized controlled trial of 202,638 women volunteers from 13 trial centres throughout Northern Ireland, Wales and England ( ISRCTN22488978). The inclusion criteria specified by the trial protocol were postmenopausal women aged between 50–74 years. The women were randomised into three groups with the ultrasound arm involving 50,639 women who underwent annual TVS examinations.

**Figure 1.**
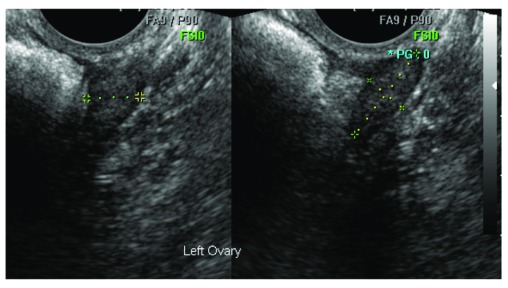
Transverse (TS) and longitudinal (LS) transvaginal ultrasound images of left ovary acquired by sonographer. This ovary was confirmed as normal and correctly measured by the expert reviewer.

Sonographers performing the examinations were required to 1) record whether the ovary had been visualized, 2) measure the ovary in 3 orthogonal dimensions, and 3) comment on its morphology. These observations were stored centrally in the Trial Management System (TMS). The sonographer measured the dimensions of each ovary using digital callipers manually positioned on the extent of the ovary boundary in static images in two orthogonal planes during the examination; see Figure 1. The distance between the calliper marks was displayed in millimeters at the bottom of the image and copied into the TMS exam record fields as D1, D2 and D3. D1 represents the longest ovarian distance in longitudinal section (LS) and D2 is the widest distance (Anteroposterior - AP) which can be measured at 90° to the line used to measure D1. The largest diameter of the ovary in transverse section (TS) is measured as D3. These dimensions allow calculation of ovarian volume using the prolate ellipsoid formula; D1xD2xD3 x0.5423.

The TVS images used to measure the ovaries for each patient were saved on the ultrasound machines at each of the 13 trial centres and periodically copied onto disks which were sent by courier to the trial coordinating centre in London where they were copied into a bespoke computer system called the Ultrasound Record Archive (URA). These archived static images allow independent confirmation as to whether the feature measured was an ovary, thus permitting a subsequent audit of the sonographer’s self-reported VR.

### Audit dataset

Sonographers who had performed >100 TVS exams between January 2008 and January 2009 and who had reported a high rate of ovary visualisation (>89%) over this period were identified. The audit dataset was created by assigning a random number to the annual exams performed by each of the sonographers during this same period and then making a random selection for each sonographer based on the value of these numbers. Inclusion criteria were both ovaries reported as visualized and the examination classified as having normal morphology. Examinations were excluded if the corresponding images were not stored in the URA. All exams audited were performed using a Medison Accuvix (model XQ, software v1.08.02, transvaginal probe type EC4-9IS 4-9 MHz).

### Audit methodology

Eight members of the UKCTOCS Ultrasound Subcommittee who were highly experienced in gynaecological scanning undertook the review. They included three consultant gynaecologists, two gynaecological radiologists and three National Health Service (NHS) superintendent grade sonographers. Originally there were nine experts but it subsequently transpired that one of the reviewers was also one of the seven sonographers being audited. Therefore, it was decided to remove this reviewer’s results from the study. Accordingly, though these experts were initially split into three groups of three, one group was reduced to two experts following the exclusion of reviewer nine.

The audit dataset was randomly split such that each group reviewed 119 exams (total 357 exams) and each expert was asked to assess 17 exams performed by each of the seven sonographers. In this way, each exam was judged by at least two separate experts. In order to avoid bias each expert was blinded as to the name of the sonographer being reviewed and the assessment of the other experts.

**Figure 2.**
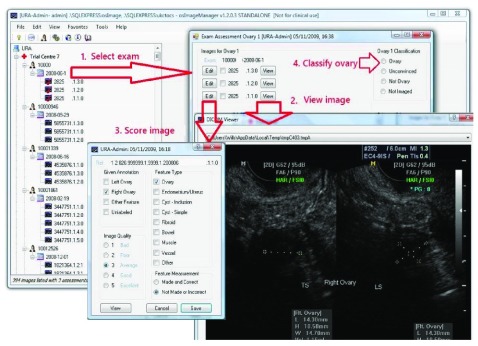
Screenshots of the osImageManager – the application used to facilitate Transvaginal Ultrasound Exam Review.

The primary aim of the audit was to confirm the self-reported visualisation of both ovaries (cVR-Both) in examinations by each of the seven sonographers, which by extension required each expert reviewer to identify the exact images used to measure both ovaries from all of the images captured during the exam (mean 5.4, range 1–30). A software tool called osImageManager was developed specifically for the reviewers ( Figure 2). It facilitated display of the images associated with each of the examinations and also recorded the review results in the audit database.

### Statistical analysis

The baseline characteristics of the women are reported by trial centre code, age, years since last period, body mass index (BMI), hysterectomy status, oral contraceptive pill (OCP) and hormone replacement therapy (HRT) use. Information from the UKCTOCS sonographer accreditation records was used to calculate the mean, range and standard deviation of their collective experience. Their level of training and qualifications was also compared. Raw confirmed VR for each sonographer, each expert and overall were calculated for left ovary (LO) and right ovary (RO) as well as jointly for both LO and RO in the same examination. However, for formal inference we calculated the confirmed VR based on a statistical model.

### Statistical modelling

All modelling was performed in Stata v14.2.

***Model description.*** The data was analysed using a bivariate probit random effects model. The bivariate outcome was the experts’ binary judgement of whether they confirmed the scan as seen or not seen, for both LO and RO. For the LO and RO portion of the model there was a scan-specific random intercept term representing the dependence of judgements within each scan, rated by three (or two) expert reviewers. The LO and RO random effects were allowed to covary as were the LO and RO error terms. In addition the model had categorical fixed effects for the original sonographer (n=7) and the expert (n=8). The details of the model can be found in Supplementary File 1. The model was fitted in Stata 14.2 with the user-written command cmp ^9^. Two additional models were fitted. Firstly, one that included the factor ‘qualification’ (gynaecologist, radiologist, sonographer) instead of the factor ‘expert’ which, fully nested within ‘qualification’, meant both terms could not be included. Secondly, the factor ‘expert’ was simply taken out for reasons described in ‘Predictions and Correlations’.

The use of this statistical model allowed us to simultaneously analyse all the data despite some scans being judged by a different number of experts. This included instances when only the LO or RO of a scan had been reviewed. By making use of model-based predictions, the model allowed us to assess the impact of each sonographer (or reviewer) whilst generalizing over the sample of reviewer (or sonographer) and volunteers, separately for LO and RO, but also for both ovaries in a joint manner. The raw proportions, summed over either sonographer or reviewer, fail to take in the within-volunteer correlation. All joint significance tests of the parameters were Wald tests.

***Predictions and correlations.*** Stata’s post-estimation command margins were used to make predictions based on the probit model parameters. Specifically, marginal probability predictions were made over the whole sample, and for each sonographer and expert for both equations (LO and RO). In addition, the joint probability of a positive outcome for both LO and RO were calculated by incorporating the estimated correlation of both the random intercepts and error terms. All marginal predictions were ‘population-averaged’ in that they were integrated over the value range of the random effects. Individual random effects were calculated using empirical Bayes means. Separate intraclass correlation coefficients (ICC) for both LO and RO were calculated using the variance component estimates (see Supplementary File 1). The ICCs estimate the dependence between the dichotomous outcomes within the same volunteer, after taking into account the fixed effects. The ICC was also calculated based on a model with no ‘expert’ term, as its inclusion will provide an ICC that reflects within-scan correlation after adjusting for each expert’s general propensity to confirm visualisation. Supplementary File 1 also describes the calculation of the correlation between the left and right ovary result for a given volunteer on a given review occasion, necessary for the joint probability estimation. Note that the correlations from a probit model are ‘tetrachoric’ – that is, the correlation of two theorised normally distributed continuous latent variables, which produce the observed binary outcomes.

## Results

An audit dataset of 357 annual TVS exams from 349 women was produced by making a random selection of 51 exams performed by each of the seven UKCTOCS sonographers who had reported ovary visualisation rates >89% for the exams they had performed during the study period (1/1/08 to 31/12/08) irrespective of outcome; normal, abnormal or unsatisfactory. However, only examinations with normal morphology reported were reviewed. Fifteen reviews were ineligible for various reasons.

The eight expert reviewers performed the image review at locations in Derby, Manchester, Bristol and London. They collectively spent approximately 100 hours conducting their audit of the work of the seven UKCTOCS sonographers. The sonographers had a mean experience of 14.5 years (range 7–23, SD 7). They operated in five different trial centres with two pairs of sonographers working in the same centre. All sonographers were accredited by UKCTOCS during 2008.

The 349 women whose exams were included in the audit dataset had a mean age of 60.0 years (range 50.2–73.3, SD 5.85), mean age at last period of 49.3 years (range 27.9–70.0, SD 5.66), mean BMI of 26.2 (range 17.5–45.1, SD 4.17), use of HRT at recruitment of 24.9%, ever use of OCP of 64.7% and a history of hysterectomy in 12.4%.

### Model results

**Figure 3.**
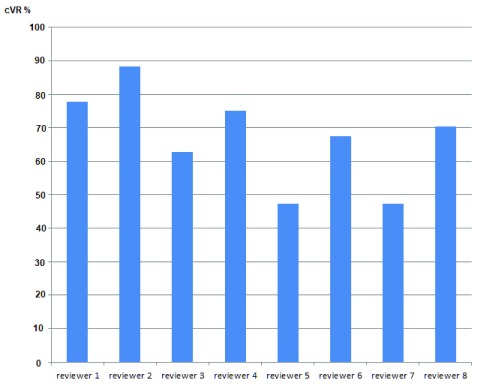
Variation in cVR-Both by individual experts as calculated by the random effects bivariate probit model.

**Figure 4.**
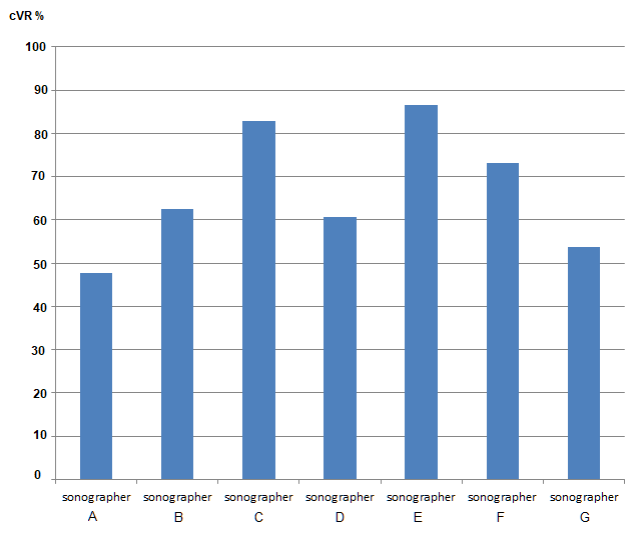
Variation in cVR-Both by individual sonographers as calculated by the random effects bivariate probit model.

In total the model fitted 1871 ultrasound scan assessments formed from 940 LO scans and 931 RO scans resulting in 945 scans where at least one ovary was included. The fixed effects of both sonographer and expert were highly significant for either left or right ovary (joint p<0.0001 always, Table 1). As expected, the fitted predictions for LO or RO separately were close to the raw proportions over the same sample (see Table 2) because the design was (largely) balanced and the predictions did not include an adjusting variable. The overall LO prediction was 0.78 (95% CI: 0.75-0.81), but by sonographer this ranged from 0.65 to 0.89. By reviewer, the range was from 0.59 to 0.93. For RO, predicted probabilities were typically higher; overall prediction was 0.80 (95% CI: 0.77-0.83), sonographer predictions ranged from 0.62 to 0.97 and reviewer predictions ranged from 0.66 to 0.94. Not all sonographer or reviewer rank orderings were the same for LO and RO, for example reviewer 7 was the lowest for LO and reviewer 5 for RO. This was in contrast to the raw proportions where reviewer 7 gave the lowest percentage of confirmations for both LO and RO. In a separate model where expert was replaced by ‘qualification’, sonographers had significantly higher confirmed VR for both LO ( *β*=0.74 95% CI: 0.38-1.10) and RO ( *β*=0.86 95% CI: 0.40-1.32) compared to gynaecologists ( Table 1). Radiologists also had higher confirmed VR than gynaecologists but this was only significant at the 5% level for LO. The mean cVR-Both obtained using the model was 67.2%, ranging from 47.6% to 86.5% (95%CI: 63.9-70.5%, Table 2) and Figure 3 and Figure 4 present marginal joint predictions (cVR-Both) for individual experts and sonographers respectively.

**Table 1.** Results of the random effects bivariate probit model – fixed and random effects.

Fixed effects						
	beta	standard error	L95% CI	U95% CI	p-value	left vs right
LEFT OVARY						
sonographer ID (vs A)					0.0000	p=0.1153
Sonographer B	0.577	0.274	0.039	1.115		
Sonographer C	1.202	0.300	0.615	1.789		
Sonographer D	0.196	0.268	-0.330	0.722		
Sonographer E	1.142	0.295	0.564	1.721		
Sonographer F	0.773	0.279	0.225	1.320		
Sonographer G	0.086	0.261	-0.425	0.597		
reviewer ID (vs reviewer 1)					0.0000	p=0.7544
reviewer 2	0.620	0.371	-0.106	1.347		
reviewer 3	-0.354	0.313	-0.968	0.261		
reviewer 4	-0.204	0.305	-0.802	0.394		
reviewer 5	-1.047	0.301	-1.636	-0.457		
reviewer 6	-0.362	0.316	-0.983	0.258		
reviewer 7	-1.130	0.291	-1.701	-0.559		
reviewer 8	-0.180	0.306	-0.781	0.421		
Qualification (vs gynaecologist) *					0.0002	p=0.313
sonographer	0.741	0.183	0.382	1.099		
radiologist	0.442	0.195	0.060	0.825		
constant	0.894	0.282	0.341	1.447	0.0020	
RIGHT OVARY						
sonographer ID (vs A)	0.484	0.318	-0.140	1.107		
Sonographer B	1.602	0.369	0.878	2.326		
Sonographer C	0.785	0.331	0.135	1.434		
Sonographer D	2.470	0.460	1.569	3.371		
Sonographer E	1.108	0.342	0.438	1.777		
Sonographer F	0.360	0.308	-0.245	0.964		
Sonographer G						
reviewer ID (vs reviewer 1)					0.0000	
reviewer 2	0.528	0.476	-0.405	1.462		
reviewer 3	-0.922	0.392	-1.691	-0.154		
reviewer 4	0.020	0.398	-0.760	0.800		
reviewer 5	-1.303	0.387	-2.060	-0.545		
reviewer 6	-0.546	0.408	-1.347	0.254		
reviewer 7	-1.182	0.367	-1.901	-0.464		
reviewer 8	-0.501	0.383	-1.252	0.250		
Qualification (vs gynaecologist) *					0.0010	
sonographer	0.861	0.236	0.399	1.320		
radiologist	0.133	0.233	-0.323	0.589		
constant	1.003	0.352	0.313	1.693	0.0040	
**Random effects** **and correlations**						p=0.4806
	estimate	standard error	L95% CI	U95% CI		
left ovary RE variance	0.758	0.217	0.332	1.183		p=0.210
right ovary RE variance	1.226	0.330	0.579	1.873		
random effect covariance	0.293	0.144	0.011	0.576		
random effect correlation	0.304	0.126	0.042	0.528		
error term correlation	0.473	0.107	0.240	0.654		
LO, RO correlation	0.387	0.064	0.262	0.513		
left ovary ICC	0.431	0.070	0.294	0.569		
right ovary ICC	0.551	0.067	0.420	0.681		
left ovary ICC **	0.396	0.068	0.264	0.529		
right ovary ICC **	0.507	0.065	0.379	0.635		

^*^from a different model that replaces 'expert' with 'specialism'

^**^ from a different model that excludes 'expert'

**Table 2.** Results of the random effects bivariate probit model – marginal predictions.

POST ESTIMATION					
Marginal predictions (population averaged)					
	probability	standard error	L95% CI	U95% CI	Raw proportion
LEFT OVARY=1					
Overall	0.776	0.015	0.747	0.806	0.774
sonographer A	0.653	0.048	0.558	0.747	0.654
sonographer B	0.787	0.041	0.707	0.867	0.780
sonographer C	0.892	0.029	0.835	0.948	0.893
sonographer D	0.702	0.047	0.610	0.793	0.729
sonographer E	0.884	0.030	0.825	0.942	0.885
sonographer F	0.825	0.036	0.753	0.896	0.817
sonographer G	0.675	0.046	0.584	0.765	0.657
reviewer 1	0.852	0.031	0.791	0.913	0.847
reviewer 2	0.932	0.022	0.888	0.975	0.930
reviewer 3	0.786	0.037	0.713	0.859	0.793
reviewer 4	0.816	0.034	0.748	0.883	0.817
reviewer 5	0.618	0.044	0.531	0.704	0.632
reviewer 6	0.784	0.038	0.710	0.858	0.784
reviewer 7	0.595	0.044	0.508	0.681	0.585
reviewer 8	0.820	0.034	0.753	0.887	0.813
RIGHT OVARY=1					
Overall	0.800	0.015	0.771	0.830	0.799
sonographer A	0.624	0.051	0.523	0.725	0.637
sonographer B	0.732	0.047	0.639	0.825	0.731
sonographer C	0.906	0.028	0.851	0.961	0.893
sonographer D	0.790	0.043	0.705	0.874	0.807
sonographer E	0.969	0.016	0.937	1.000	0.971
sonographer F	0.843	0.037	0.771	0.915	0.825
sonographer G	0.706	0.047	0.614	0.797	0.704
reviewer 1	0.883	0.028	0.828	0.938	0.890
reviewer 2	0.935	0.022	0.892	0.978	0.939
reviewer 3	0.735	0.040	0.657	0.813	0.750
reviewer 4	0.885	0.027	0.833	0.938	0.870
reviewer 5	0.655	0.042	0.572	0.738	0.675
reviewer 6	0.804	0.036	0.733	0.875	0.810
reviewer 7	0.682	0.040	0.603	0.760	0.644
reviewer 8	0.812	0.035	0.744	0.879	0.812
JOINT (LO=1, RO=1)					
Overall	0.672	0.017	0.639	0.705	0.670
sonographer A	0.476	0.047	0.384	0.569	0.516
sonographer B	0.626	0.047	0.534	0.718	0.618
sonographer C	0.828	0.035	0.760	0.895	0.814
sonographer D	0.606	0.047	0.514	0.698	0.621
sonographer E	0.865	0.031	0.804	0.927	0.863
sonographer F	0.730	0.041	0.649	0.812	0.714
sonographer G	0.538	0.046	0.448	0.628	0.515
reviewer 1	0.779	0.035	0.711	0.847	0.780
reviewer 2	0.883	0.028	0.828	0.937	0.886
reviewer 3	0.627	0.040	0.548	0.706	0.612
reviewer 4	0.751	0.036	0.681	0.822	0.730
reviewer 5	0.473	0.040	0.394	0.552	0.470
reviewer 6	0.674	0.040	0.595	0.752	0.672
reviewer 7	0.473	0.040	0.394	0.551	0.492
reviewer 8	0.704	0.038	0.630	0.779	0.723

The variance estimates for the LO and RO random effects were 0.76 and 1.23 respectively ( Table 1), but this did not differ statistically (p=0.210). Indeed, despite the observed differences, there was no statistical difference in the LO versus RO effects concerning sonographer (p=0.115), reviewer (p=0.754) or the model as whole (p=0.481). The correlation of the LO and RO ovary random effects was 0.30 (95% CI: 0.04-0.53) and the error term correlation was 0.47 (95% CI: 0.24-0.65), implying a correlation of 0.39 (95% CI: 0.26-0.51) for the paired outcome of LO and RO for a given volunteer and occasion. This compares to the tetrachoric correlation of raw data of 0.51, and to 0.37 when the fixed effects are included in a standard bivariate probit model. The resultant within-volunteer correlation (ICC) for the repeated outcomes within a volunteer were 0.43 (95% CI: 0.29-0.57) and 0.55 (95% CI: 0.42-0.68) for LO and RO respectively. In addition, the ICCs for a model excluding the mean effect of the ‘expert’ term, were lower at 0.40 (95% CI: 0.26-0.53) for LO and 0.51 (95% CI: 0.38-0.64) for RO.

DataKey.txt – description of data fields; UKCTOCS TVC audit data biprobit format-0.csv; UKCTOCS TVC audit data biprobit format-0.dta; UKCTOCS TVC audit data do file.do Click here for additional data file. Copyright: © 2018 Stott W et al. 2018 Data associated with the article are available under the terms of the Creative Commons Zero "No rights reserved" data waiver (CC0 1.0 Public domain dedication).

## Discussion

Our audit suggests that sonographer’s self-reported visualization rates of postmenopausal ovaries they judged to have normal morphology is unreliable. Our study was facilitated by the unique TMS and URA systems employed in UKCTOCS which permitted a retrospective review of the images and measurements recorded by the sonographer. It could be argued that the static images used for this audit represent a snapshot of a continuous pelvis examination so might not truly represent what was seen by the sonographer. Nevertheless, these static images were used to measure the ovaries, so the structure marked by the callipers was definitely considered to be an ovary by the sonographer.

We analysed the data using a statistical model that accounted for the correlated structure of the data, between left and right ovary scans, and between the same scan viewed by the experts. Normality was assumed for the underlying latent variable (‘propensity to confirm visualisation’) and for the distribution of the ovary-specific volunteer random effects. The model gave predictions in the probability scale that different only slightly from the raw proportions, due to the nature of the study design. One clear benefit to using a statistical model with random effects is that all the data could be analysed together, and producing variance component estimates that allow the calculation of ICCs. The value of the ICC was higher for the right ovary then left, though not significantly different, and for both were modest: 0.40 for LO and 0.51 for RO when excluding the expert term from the fixed effects, the only variable that varied over each scan’s repeated assessments. Hence the ICC is a measure of inter-rater (expert) agreement, and suggests that although there is moderate concordance, the experts cannot be relied upon to replicate the judgements of each other. However, such lack of agreement in respect of each individual scan does not change of the overall conclusion of the audit in terms of the unreliability of the sonographer’s self-reported visualization rates.

We have previously reported on the Quality Control (QC) of UKCTOCS TVS scanning with similar exam selection criteria (ovaries were seen and normal) ^7^. A single expert reviewed 1000 randomly chosen TVS examinations which had been performed by 96 sonographers. The expert’s cVR-Both was 50% compared to the 100% VR as self-reported by the sonographers for these examinations. This result is broadly consistent with the results reported in this study for the group of seven sonographers with mean cVR-Both of 67.2%. The significant variation in cVR-Both across sonographer of normal postmenopausal ovaries is probably due to differences in sonographer ability and the subjective nature of this examination; a supposition supported by findings reported by Sharma *et al.* ^8^.

## Limitations of the study

Intra-observer reproducibility was not addressed so the capability of individual experts to provide consistent results for the same exams was not measured. The study design was generally balanced, and potential confounders that might possibly affect visualization should be expected to be evenly distributed across experts due to the randomization process. However, it is conceivable that these confounders may not be balanced across sonographers, due to potential geographical differences in their distribution. This was not a major concern, but the factors could have been seamlessly absorbed into the model and produced sonographer predictions conditional on equal covariate distribution.

## Conclusion

The results of this audit confirm that the visualization of postmenopausal normal ovaries by seven ‘high performing’ sonographers, as assessed by eight experts, could not be considered reliable given that in almost a third of their examinations structures other than an ovary had been mistakenly measured in at least one of the ovaries. However, individual sonographer performance varied significantly from 47% to 87% cVR-Both. These results show that it is possible for some sonographers to correctly visualize both ovaries when scanning a range of menopausal women so raising the possibly that other sonographers might achieve similar results if supported by a suitable quality improvement programme.

This audit highlights the problem of sonographers routinely mistaking other features like the bowel as ovaries when scanning postmenopausal women. It also highlights the difficulties of providing effective Quality Control (QC) for such scans in a large scale screening programme. Specifically, it shows that undertaking the type of expert review conducted by this study for a substantial number of sonographers on a regular basis would not be feasible without creating dedicated teams specializing in normal ovary identification from TVS images of postmenopausal women. Therefore there is a need for further research to explore how independent and reliable QC metrics for TVS might be obtained by other means, for example by the automated analysis of TVS scan images both static and video. Recent advances in machine learning research, particularly in the area of deep neural networks, suggest it might soon be viable to construct a system able to determine sonographer VR from a collection of images captured during a series of TVS examinations. Indeed, the use of such deep learning techniques in the gathering of quality metrics from obstetric ultrasound images is already reporting some promise ^10^.

The work done by the UKCTOCS group on the QC of TVS scanning seeks to improve understanding of challenges associated with performing screening for ovarian cancer on a large scale and at multiple centres. All previous studies of ultrasound screening of postmenopausal ovaries for the early detection of cancer (excepting the recent QC study by our group) have accepted the self-reporting of ovarian visualisation rates as accurate. This is the first published audit of self-reporting of ovarian visualization rates and the results cause us to question the reliability of this metric, particularly for QC purposes.

## Ethics approval

The UKCTOCS study was approved by North West Multicentre Research Ethics Committee 21/6/2000; MREC reference 00/8/34. It is registered as an International Standard Randomised Controlled Trial (no. ISRCTN22488978).

## Data availability

The data referenced by this article are under copyright with the following copyright statement: Copyright: © 2018 Stott W et al.

Data associated with the article are available under the terms of the Creative Commons Zero "No rights reserved" data waiver (CC0 1.0 Public domain dedication). 

Dataset 1: DataKey.txt – description of data fields; UKCTOCS TVC audit data biprobit format-0.csv; UKCTOCS TVC audit data biprobit format-0.dta; UKCTOCS TVC audit data do file.do. DOI, 10.5256/f1000research.15663.d213048 ^11^

Stata v14.2 was used in conjunction with the files in Dataset 1 to obtain the results presented in this paper.

## Supplementary material

**Supplementary File 1: Description of the probit random effects model.** Specification of the probit random effects model and details of methods used for calculating correlations and predictions as referenced in the Statistical Modelling section of the Methods part of the paper.
