# Supplementary material for: Audit of transvaginal sonography of normal postmenopausal ovaries by sonographers from the United Kingdom Collaborative Trial of Ovarian Cancer Screening (UKCTOCS)
Source: F1000Res. 2018 Aug 10;7:1241. [Version 1] doi: 10.12688/f1000research.15663.1 (PMC6173132; doi:10.12688/f1000research.15663.1)
Supplement: Supplementary file 2 [file f1000research-7-17090-s0001.tgz › be95b916-aa3b-40b1-9211-31299d7d6d0f.docx]

**Supplementary File 1**

**Model specification**

For a review of a volunteer’s scan the probability of left (*L*) and right ovary (*R*) being confirmed as ‘seen’ is modelled by a bivariate random effects probit model:

$$\Pr\left( y_{ijL}=1, y_{ijR}=1 \right|\boldsymbol{x}_{ijk},\zeta_{ik})=\Phi_{2}({XB}^{L}, {XB}^{R};\rho)$$

where $\Phi_{2}(., .;\rho)$ denotes the cumulative distribution function of the bivariate standard normal distribution, ${XB}^{k}$ is the linear portion of the model including random effectsand $\rho$ is the correlation between latent error terms. A probit model can be characterized by an underlying latent variable ${y^{*}}_{ijk}$ (here the propensity for an expert to confirm a sonographer’s positive scan) and so the observed outcome is defined as:

$y_{ijk}=1 if$ ${y^{*}}_{ijk}>0$

$y_{ijk}=0 if$ ${y^{*}}_{ijk}\leq0$

and the linear portion of the model is:

$${y^{*}}_{ijk}=\beta_{0k}+\beta_{1k}x_{1ijk}+\ldots\beta_{zk}x_{zijk}+\zeta_{ik}+\varepsilon_{ijk}$$

In more detail $y_{ijk}$ is the outcome of scan review for volunteer *i* (1, …, 354) viewed on occasion *j* (1, …, 3) looking at ovary *k* (*L*=left, *R*=right), where the outcome *y*=0 is not confirmed or *y*=1 confirmed by reviewer, $x_{aijk}$ is the value for the *a*^th^ (1,…*z*) covariate (fixed effect) for volunteer *i* viewed on occasion *j* looking at ovary *k*. Categorical variables in the model are reviewer (7 parameters) and sonographer (6 parameters)

$\beta_{ak}$is the parameter value for the corresponding *a*^th^ covariate for the *k*^th^ equation

$\zeta_{iL}, \zeta_{iR}$ are the correlated random intercepts for volunteer left ovary and volunteer right ovary respectively.

The vector of random intercepts is allowed to covary but is independent from the covariates and the error terms, and normally distributed:

$$\left[ \begin{matrix} {\zeta_{iL} \atop\zeta_{iR}} \end{matrix} \right]\sim N\left( \left[ \begin{matrix} 0 \\ 0 \end{matrix} \right],\left[ \begin{matrix} \psi_{LL} & \psi_{LR} \\ \psi_{LR} & \psi_{RR} \end{matrix} \right] \right)$$

The error terms for $y^{*}$ of each equation *k* (for the same volunteer *i* and same occasion *j*) are also correlated:

$$\left[ \begin{matrix} {\varepsilon_{ijL} \atop\varepsilon_{ijR}} \end{matrix} \right]\sim N\left( \left[ \begin{matrix} 0 \\ 0 \end{matrix} \right],\left[ \begin{matrix} 1 & \rho\\ \rho& 1 \end{matrix} \right] \right)$$

**Correlations**

The intraclass correlation coefficient (ICC) for ovary *k* was calculated using the probit model-based formula:

$$\rho^{[k]} =\frac{\psi_{kk}}{\psi_{kk}+1}$$

The correlation between the left and right ovary result for a given volunteer on a given review occasion is:

$$\rho^{*} =\frac{\psi_{LR}+\rho}{\surd(\psi_{LL}+1)\surd(\psi_{RR}+1)}$$

confidence intervals were derived using the Stata command nlcom which allows for non-linear combinations of estimated coefficients.

**Predictions**

Unfortunately, cmp does not currently offer population-averaged predictions whereby the prediction is based on integrating over the range of the random effect. Instead, it allows only fixed mean predictions, meaning just the fixed portion of the linear predictor is included and one must assume that the prediction is for a subject with a zero value for the random effect(s). In order to obtain these marginal predictions we were able to refit the model using Stata command gsem (for generalized structural equation modelling). However, a bivariate probit model fitted with this command results in rescaled estimates and standard errors.

To obtain the joint probability of a positive outcome for both left and right ovary we utilized the ability of the margins command to generalize the prediction to any function of the standard predictions allowed using the expression() option. Specifically, the expression included Stata’s own cumulative distribution function of the bivariate standard normal binorm, with the population-averaged linear prediction for left and right ovary and correlation $\rho^{*}$ (see above) plugged into the full expression. Note the $\rho^{*}$ term was represented as the function of the individual variance estimates using their official Stata legend names, rather than a simple scalar, in order to incorporate the appropriate level of uncertainty in the confidence intervals.
