# Supplementary material for: Audit of transvaginal sonography of normal postmenopausal ovaries by sonographers from the United Kingdom Collaborative Trial of Ovarian Cancer Screening (UKCTOCS)
Source: F1000Res. 2018 Aug 10;7:1241. [Version 1] doi: 10.12688/f1000research.15663.1 (PMC6173132; doi:10.12688/f1000research.15663.1)
Supplement: Peer review history

# Peer review: Referee response for version 1

**DOI:** 10.5256/f1000research.17090.r38129

**Published:** 2018-10-03

**Authors:** Christine D. Berg

**Affiliations:** 1 National Cancer Institute (NCI), U.S. Department of Health and Human Services, National Institutes of Health (NIH), Bethesda, MD, USA

The United Kingdom Collaborative Trial of Ovarian Cancer Screening was a high-quality randomized clinical trial evaluating trans-vaginal ultrasound screening (TVUS) compared to a multi-modal screening (MMS) with Risk of Ovarian Cancer Algorithm (ROCA) testing based on CA-125 measurements followed by ultrasound when indicated compared to no screening in 202,546 women of average risk of ovarian cancer.  After 7 to 11 rounds of screening and up to 14 years of follow-up results trended toward benefit with ultrasound (HR = 0.91 [95% CI, 0.76 to 1.09]) and MMS (HR = 0.89 [95% CI, 0.74 to 1.08]) ^1^. A comprehensive quality assurance program for TVUS was undertaken ^2^. 

 This current paper is the result of an audit ordered by the trial’s Ultrasound Management Subcommittee of seven sonographers reporting rates of visualizing both ovaries of > 89% after an accreditation program done by UKCTOCS in 2008.  Eight experts reviewed 357 archived, static images centrally stored that also had measurement markers recording ovary dimensions.  The mean visualization rate upon review for both ovaries fell to 67.2% with a range of 47.6 % to 86.5%.  The range between the experts was 47.3% to 88.3%.

 The trialists are to be commended for the design and conduct of this impressive ovarian cancer screening trial which shows some evidence of stage-shift and mortality reduction but after many rounds and long follow-up in a large group of women.  The quality assurance plans in place to train and monitor TVUS results is impressive.  While the technology dates to 2008-2009 and highly-selected experts might do better, the results of this audit strike a cautionary note about the rate of probably unavoidable non-visualization of ovaries with TVUS in post-menopausal women.  They call for expanded efforts in automated analysis with machine learning.  Improved technologies or biomarkers are also needed to realize the promise of lowering ovarian cancer mortality with early detection.

I have read this submission. I believe that I have an appropriate level of expertise to confirm that it is of an acceptable scientific standard.

## References

[1] JacobsIMenonURyanAGentry-MaharajABurnellMKalsiJAmsoNApostolidouSBenjaminECruickshankDCrumpDDaviesSDawnayADobbsSFletcherGFordJGodfreyKGunuRHabibMHallettRHerodJJenkinsHKarpinskyjCLeesonSLewisSListonWLopesAMouldTMurdochJOramDRabideauDReynoldsKScottISeifMSharmaASinghNTaylorJWarburtonFWidschwendterMWilliamsonKWoolasRFallowfieldLMcGuireACampbellSParmarMSkatesS: Ovarian cancer screening and mortality in the UK Collaborative Trial of Ovarian Cancer Screening (UKCTOCS): a randomised controlled trial. *The Lancet*.2016;387(10022) : 10.1016/S0140-6736(15)01224-6 945-956 10.1016/S0140-6736(15)01224-6 PMC477979226707054

[2] SharmaABurnellMGentry-MaharajACampbellSAmsoNNSeifMWFletcherGBrunellCTurnerGRangarRRyanAJacobsIMenonU: Quality assurance and its impact on ovarian visualization rates in the multicenter United Kingdom Collaborative Trial of Ovarian Cancer Screening (UKCTOCS). *Ultrasound Obstet Gynecol*.2016;47(2) : 10.1002/uog.14929 228-35 10.1002/uog.14929 26095052PMC4755159

# Peer review: Referee response for version 1

**DOI:** 10.5256/f1000research.17090.r38131

**Published:** 2018-09-21

**Authors:** Mark E. Sherman

**Affiliations:** 1 Department of Health Sciences Research (HSR), Mayo Clinic‎, Jacksonville, FL, USA

This manuscript assesses the retrospective findings of 8 expert reviewers in assessing ultrasounds reported by 7 ultrasonographers who recorded visualization of both normal postmenopausal ovaries in >89% of scans in the United Kingdom Collaborative Trial of Ovarian Cancer Screening (UKCTOCS). The study set included 357 examinations from 349 women and 1,871 randomly chosen scans reviewed by these ultrasonographers selected from a one-year period in the trial. The main aim was to assess whether the interpretation of bilateral visualization of normal ovaries is reliable, comparing original reports to retrospective expert review. Data were analyzed using a bivariate probit random effects model with outcomes of bilateral visualization versus not.

 Notably, normal postmenopausal ovaries may be quite small and the average age of women in UKCTOCS was about 60 years. The data show poor reliability across measures: bilateral visualization for original reviewers showed a mean of 67%, with a range of 47.6%-86.5%; expert results ranged from 47.3%-88.3%. Agreement among expert reviewers was also modest. The authors conclude that further research is needed to develop reliable quality control metrics for transvaginal ultrasound.

 It is unclear how much expert reviewers knew about methods for assembling the study set and other details, which may have influenced interpretations. The study did not include a random sample of scans from the trial to serve as “distractors” or to provide a reference for comparison, and reliability data were not compared with external standards, such as measurements of ovaries that may have been removed later, CA 125 levels or rare cancer outcomes. External reviewers who were uninvolved with UKCTOCS may be of interest and possibly could be achieved via a web-based approach, at least for a subset.

 This study in combination with prior reports from UKCTOCS (e.g. Stott et al and Sharma et al) provides a composite picture of the performance of ultrasound in the trial; however, the generalizability of the current study is difficult to assess, given the unusual method of scan selection and the engagement of reviewers who were intimately knowledgeable about the trial and perhaps aware of the design of this project. Irrespective of these concerns, the data from UKCTOCS suggest that ultrasound of normal ovaries among older women has limitations.

 Given that reviewers were experienced and specifically trained for the task at hand, there are additional unknowns about whether and how performance could be improved, and how much  of reviewers’ performances reflect inherent limitations of ultrasound for assessment of ovaries and ovarian cancer screening.  Bodelon et al ^1^ reported a high frequency of non-visualization of ovaries in the Prostate, Lung, Colorectal, and Ovarian (PLCO) screening trial, with a tendency for individual women to have repeated non-visualization. Further, although non-visualization is likely a marker of smaller ovarian size on average, it is notable that non-visualization conferred at best a marginally reduced risk of developing ovarian cancer in PLCO. Analysis of serial ovarian volumes in PLCO suggested that enlargement occurs rapidly within one to two years of cancer detection, and therefore, would be unlikely to have meaningful impact on clinical outcomes.

 In a narrow sense, if ultrasound is to be used for ovarian cancer screening, then a better quality control metric than the frequency with which ovaries are visualized is needed. In a broader sense, this study and related literature call into question whether ultrasound imaging is useful in ovarian cancer screening, especially for high-grade serous carcinomas. To date, ovarian cancer screening and CA-125 has failed to achieve a reduction in ovarian cancer mortality. Although unproven, growing evidence points to the origin of many high-grade serous carcinomas, the most frequent lethal type of ovarian cancer, from the distal fallopian tube (fimbria), rather than from the ovarian surface epithelium. In contrast, other ovarian cancers (i.e. endometrioid and clear cell) may arise from endometriosis in the ovary and tend to remain organ confined for lengthier periods (present as stage I). Animal models of tubal cancer have shown that spread to the ovaries may accelerate disease progression (Perets et al ^2^), but many questions about the pathogenesis of serous cancers among women, including the sojourn time of disease development and the role of the ovary in promoting metastatic spread. These fundamental questions raise larger issues about the role of assessing the ovary as part of cancer screening and the potential of ultrasound to identify cancers at early curable stage. Greater knowledge of the pathogenesis of ovarian cancer, especially high-grade serous cancers, may pose a barrier to improved early detection. Larger issues could be addressed to place the results of the study in context.

I have read this submission. I believe that I have an appropriate level of expertise to confirm that it is of an acceptable scientific standard, however I have significant reservations, as outlined above.

## References

[1] BodelonCPfeifferRMBuysSSBlackAShermanME: Analysis of serial ovarian volume measurements and incidence of ovarian cancer: implications for pathogenesis. *J Natl Cancer Inst*.2014;106(10) : 10.1093/jnci/dju262 10.1093/jnci/dju262 25217774PMC4168310

[2] PeretsRWyantGAMutoKWBijronJGPooleBBChinKTChenJYOhmanAWStepuleCDKwakSKarstAMHirschMSSetlurSRCrumCPDinulescuDMDrapkinR: Transformation of the fallopian tube secretory epithelium leads to high-grade serous ovarian cancer in Brca;Tp53;Pten models. *Cancer Cell*.2013;24(6) : 10.1016/j.ccr.2013.10.013 751-65 10.1016/j.ccr.2013.10.013 24332043PMC3917315

# Response

**Published:** 2018-10-01

**Authors:** Will Stott

**Affiliations:** University College London, Institute for Women's Health, UK

We thank Prof Sherman for his review, but seek clarification so that we might improve our paper. We note that he does not consider our conclusions are adequately supported by our results. **Does he believe our results show that **rep**orts from an individual sonographer about the ovary visualisation of her own scans  produce more reliable quality control metrics** **than the combined judgement of a team of eight experts **when the reviewing the images the sonographer used to measure ovaries as taken from a random sample her scans over a year? If so, we should be grateful if he would identify the data in our results that supports such a conclusion.

# Peer review: Referee response for version 1

**DOI:** 10.5256/f1000research.17090.r37084

**Published:** 2018-08-28

**Authors:** John R. van Nagell, Frederick Ueland, Edward Pavlik

**Affiliations:** 1 Department of Obstetrics and Gynecology, Division of Gynecologic Oncology, Markey Cancer Center (MCC), University of Kentucky, Lexington, KY, USA; 2 Division of Gynecologic Oncology, Department of Obstetrics and Gynecology, University of Kentucky, Lexington, KY, USA

This paper reports the audit results of seven sonographer self-reporting high visualization rates of normal postmenopausal ovaries in the United Kingdom Collaborative Trial of Ovarian Cancer Screening (UKCTOCS). Eight experts reviewed static images from 357 ultrasound examinations performed on 349 postmenopausal women (mean age 60.0 yrs) to assess whether visualization of both ovaries with normal morphology could be confirmed. A random effects bivariate probit model was fitted to analyze the results. Both normal ovaries could be visualized in only two-thirds of cases, in both the sonographer and expert groups, and there was variation between findings reported by sonographers and their expert reviewers. As a result, the authors conclude that self-reported visualization of normal ovaries by sonographers is unreliable. The authors further suggest that effective quality control in the interpretation of large numbers of ovarian ultrasound images is difficult, and may be enhanced by applying recent advances in machine-learning to this problem. 

 This audit confirms that the ultrasound evaluation of normal postmenopausal ovaries is often challenging because of their low volume. In fact, ovarian volumes continue to decrease after menopause, so ultrasound visualization may be even more difficult in women in the seventh and eighth decades of life ^1^. As demonstrated, interpretation of ovarian ultrasound images is subjective, and may vary significantly between sonographers and expert physicians. For this reason, ultrasound scans, both of normal and abnormal ovaries, are reviewed by physicians for all women enrolled in the University of Kentucky Ovarian Screening Trial ^2^. This review is time-consuming, however, and may be impractical as ovarian cancer screening is made available to large populations of at-risk women. The authors are to be commended for supporting efforts to adapt machine-learning technology to achieve automated analysis of static and video ultrasound images. 

 A related and equally important issue is the ability of sonographers and expert reviewers to identify those morphologic patterns associated with early ovarian cancer. The evaluation algorithm used to identify those ultrasound patterns associated with ovarian cancer is important because it increases the positive predictive value of screening, and limits operative intervention for benign disease. We agree with the authors that ovarian cancer screening should be offered to women at moderate risk for ovarian cancer as a method to reduce disease mortality through early detection ^3^, and applaud their efforts to standardize ultrasound interpretation.

We have read this submission. We believe that we have an appropriate level of expertise to confirm that it is of an acceptable scientific standard.

## References

[1] PavlikEJDePriestPDGallionHHUelandFRReedyMBKryscioRJvan NagellJR: Ovarian volume related to age. *Gynecol Oncol*.2000;77(3) : 10.1006/gyno.2000.5783 410-2 10.1006/gyno.2000.5783 10831351

[2] van NagellJRMillerRWDeSimoneCPUelandFRPodzielinskiIGoodrichSTElderJWHuangBKryscioRJPavlikEJ: Long-term survival of women with epithelial ovarian cancer detected by ultrasonographic screening. *Obstet Gynecol*.2011;118(6) : 10.1097/AOG.0b013e318238d030 1212-21 10.1097/AOG.0b013e318238d030 22105249

[3] MenonUKarpinskyjCGentry-MaharajA: Ovarian Cancer Prevention and Screening. *Obstet Gynecol*.2018;131(5) : 10.1097/AOG.0000000000002580 909-927 10.1097/AOG.0000000000002580 29630008
